# Diabetes and Insulin Therapy, but Not Metformin, Are Related to Hepatocellular Cancer Risk

**DOI:** 10.1155/2015/570356

**Published:** 2015-05-13

**Authors:** Luca Miele, Cristina Bosetti, Federica Turati, Gianlodovico Rapaccini, Antonio Gasbarrini, Carlo La Vecchia, Stefania Boccia, Antonio Grieco

**Affiliations:** ^1^Institute of Internal Medicine, Catholic University of Rome, Largo Francesco Vito 1, 00168 Rome, Italy; ^2^Internal Medicine and Gastroenterology Unit, Complesso Integrato Columbus, Via Giuseppe Moscati 31-33, 00168 Rome, Italy; ^3^Department of Epidemiology, IRCCS-“Mario Negri” Institute for Pharmacological Research, Via G. La Masa 19, 20156 Milan, Italy; ^4^Department of Clinical Sciences and Community Health, University of Milan, Via G. Venezian 1, 20122 Milan, Italy; ^5^Institute of Public Health Section of Hygiene, Catholic University of Rome, Largo Francesco Vito 1, 00168 Rome, Italy

## Abstract

*Introduction*. Metabolic conditions, including type 2 diabetes, have been related to hepatocellular carcinoma (HCC) risk. We have further analyzed the role of diabetes and antidiabetic treatments on HCC. *Methods*. Data derived from a hospital-based case-control study (Italy, 2005–2012) on 224 HCC patients and 389 controls. Odds ratios (ORs) were estimated using multiple logistic regression models. *Results*. Sixty-nine (30.9%) cases versus 52 (13.5%) controls reported a diabetes diagnosis, corresponding to a multivariate OR of 2.25 (95% confidence interval, CI = 1.42–3.56). A stronger excess risk emerged for a longer time since diabetes diagnosis (OR = 2.96 for <10 years and 5.33 for ≥10 years). Oral therapies were inversely, though not significantly, related to HCC risk, OR being 0.44 for metformin and 0.88 for sulfonylureas; conversely, insulin was nonsignificantly directly associated (OR = 1.90). Compared to nondiabetic subjects who were never smokers, those who were diabetics and ever smokers had an OR of 6.61 (95% CI 3.31–13.25). *Conclusion*. Our study confirms an over 2-fold excess HCC risk in diabetics, with a stronger excess risk in diabetic subjects who are also tobacco smokers. Metformin may decrease the risk of HCC, whereas insulin may increase the risk.

## 1. Introduction

Liver cancer is the seventh most common cancer and the third cause of cancer deaths worldwide [1, 2]. In Italy, 2950 deaths from liver cancer were registered in 2008 in men (3% of all cancer deaths) and 1500 (2% of all cancer deaths) in women [3].

Hepatocellular carcinoma (HCC) is the most common histological type of liver cancer, accounting for up to 85% of cases [4]. The major recognized factors for HCC are chronic hepatitis B virus (HBV) and hepatitis C virus (HCV) infections, heavy alcohol drinking, and tobacco smoking [4–8].

Several epidemiological studies have indicated that type 2 diabetes mellitus is also associated with an increased risk of HCC [9–15]. A meta-analysis of epidemiological studies published up to February 2011 reported a significant relative risk (RR) of 2.4 from 17 case-control studies, 2.2 from 25 cohort studies, and a RR of 2.4 from 7 cohort studies on mortality from HCC [13]. A significant trend with duration of diabetes was observed in 6 studies, with a RR of 3.3 for 10 years since diabetes diagnosis. Moreover, the presence of diabetes has been associated with metastatic HCC and with worse prognosis [16, 17].

Although data on the role of drugs for the treatment of type 2 diabetes in HCC are limited, there are indications that metformin, sulfonylureas [18], and thiazolidinediones [19] are associated with a reduced risk of HCC, whereas insulin may increase the risk [20].

We have further analyzed the role of diabetes and antidiabetic treatments in HCC risk using data from a hospital-based case-control study conducted in Italy.

## 2. Materials and Methods

### 2.1. Selection of Cases and Controls

Study subjects were recruited among patients admitted to the Teaching Hospital “Agostino Gemelli” of the Università Cattolica del Sacro Cuore, Rome, Italy, from January 2005 until July 2012, and eligibility was restricted to Caucasian individuals born in Italy. In particular, cases were 224 patients with HCC recruited among subjects referred to the Outpatient Liver Unit of the hospital. The diagnosis of HCC was performed according to the AASLD guidelines [21]. Controls were 389 patients from the same hospital, enrolled during the same time period. Around 50% of the controls were outpatients, and the remaining were patients undergoing surgical interventions (laparoscopic cholecystectomy, appendicitis, and inguinal hernia) or admitted for a wide spectrum of other acute nonneoplastic diseases. The study was conducted according to the Declaration of Helsinki and was approved by the Ethical Committee of Università Cattolica del Sacro Cuore. A written informed consent was obtained from all study subjects.

### 2.2. Data Collection

HCC cases and controls were interviewed by trained interviewers using a structured questionnaire to collect information on demographics, lifestyle habits (including tobacco smoking and alcohol drinking), and medical history. In particular, the questionnaire included information on history and duration of diabetes and use of antidiabetic drugs (metformin, sulfonylureas, and insulin). Questions focused on the time period ending one year prior to diagnosis for cases and on the year prior to the interview date for controls.

### 2.3. Data Analysis

Odds ratios (ORs) for HCC and their corresponding 95% confidence intervals (CI) were estimated using multiple logistic regression models [22] including terms for age, sex, tobacco smoking, and alcohol drinking. To test for multiplicative interaction between diabetes and tobacco smoking, we compared the difference in −2 log likelihood of the models with and without an interaction term with the *χ* ^2^ distribution with one degree of freedom. Moreover, we tested for additive interaction using the relative excess risk due to interaction (RERI) and the synergy index (*S*) [23] and tested their significance using the delta method [24].

## 3. Results

Table 1 shows the distribution of HCC cases and corresponding controls according to selected factors. Cases were somewhat older than controls, more frequently of male sex and ever smokers. History of hepatitis was reported by 71% of cases and 3% of controls.

The distribution of HCC cases and controls and corresponding ORs according to history of diabetes and use of antidiabetic drugs are given in Table 2. Sixty-nine (30.9%) cases versus 52 (13.5%) controls reported a diagnosis of diabetes, corresponding to a multivariate OR of 2.25 (95% CI = 1.42–3.56). A stronger excess risk was observed for a longer time since diagnosis of diabetes (OR = 2.96 for <10 years and 5.33 for ≥10 years). Subjects using oral antidiabetic drugs had a nonsignificantly reduced HCC risk as compared to nonusers: the OR was 0.51 for use of any oral antidiabetic drug, 0.44 for metformin, and 0.88 for sulfonylureas. Conversely, subjects using insulin had a nonsignificantly higher risk of HCC as compared to nonusers (OR = 1.90).

The combined effect of diabetes and tobacco smoking on HCC risk is shown in Table 3. Compared to nondiabetic subjects who were never smokers, those who were diabetics and ever smokers had an OR of 6.61 (95% CI = 3.31–13.25). The interaction was significant on an additive scale (RERI = 4.27, *P* = 0.052 and *S* = 4.15, *P* = 0.013), and also borderline significant on a multiplicative one (*χ* ^2^ = 3.26; *P* = 0.071).

## 4. Discussion

In this study, subjects with diabetes have an over 2-fold excess risk of HCC, in line with the evidence from several other case-control and cohort studies [11–13]. Further, HCC risk was higher in subjects with a longer history of diabetes. Again this is in agreement with the evidence from a few other investigations [13, 20, 25].

The mechanistic role of diabetes in HCC cancerogenesis is not straightforward. Diabetes may be a consequence of chronic liver diseases (such as cirrhosis, nonalcoholic fatty liver disease, and steatosis) [26, 27]. However, the persistence of an excess risk many years since diagnosis suggests that diabetes may be a real cause of HCC. Hyperinsulinemia and insulin resistance, which characterize diabetes, and the consequent upregulation of the insulin-like growth factor-1/insulin receptor substrate-1 system, may also explain the association between diabetes and HCC [28, 29]. Changes in the hepatic activity and mitosis related to metabolic alterations or to impaired liver function in diabetics are other possible explanations of the association observed [30].

Our study suggests that use of oral antidiabetic drugs, in particular metformin, reduces the risk of HCC, while insulin is associated with an increased risk. A few other investigations found an inverse association with metformin [31–34] and a direct one with insulin [35–37] and also sulfonylureas [31, 32]. This is in line with the observation that metformin reduces insulin resistance, suppresses tumour formation, and inhibits cell growth, while insulin may promote carcinogenesis [38, 39]. However, potential confounders, such as the severity of diabetes and the variable baseline characteristics of patients using different drugs may influence the association between antidiabetic drugs and the subsequent risk of developing HCC.

Cases and controls in our study came from comparable catchment areas, were interviewed by uniformly trained interviewers, and were unaware of a possible link between diabetes and liver cancer, thereby reducing the likelihood of potential selection and recall bias. Our study has however some limitations. First, our questionnaire recorded frequency of alcohol consumption one year before cancer diagnosis (for cases) or interview date (for controls), and we have no information on duration of use or lifetime alcohol intake. Thus, alcohol information was not interpretable in our database due to frequent cessation of alcohol consumption in subjects with hepatitis and other chronic liver diseases. Consequently, we used information on alcohol only as a covariate for multivariate analysis, though we recognize that this may have led to some underadjustment. In the absence of allowance for alcohol, the OR for diabetes was 2.46 (95% CI 1.60–3.79), thus suggesting that even more accurate allowance for alcohol is unlikely to largely or totally explain the association with diabetes. Anyway, alcohol consumption was moderately associated with liver cancer [40]. Second, information on hepatitis was valid in cases, since it was based on third generation immunoassay; this does not apply to all controls, though the almost 3% prevalence of hepatitis is in broad agreement with data from the Italian general population [5]. When we added a term for hepatitis to the model, the OR for diabetes became 4.75 (95% CI, 2.54–8.86). This indicates that the association between diabetes and HCC cannot be explained by a greater frequency of HBV/HCV infections among diabetic subjects. The increase of the RR is due to the fact that no control with hepatitis reported a history of diabetes. Thus, the models including a term for hepatitis were largely unstable and consequently were not considered in the main analyses. Third, in our models, we were unable to allow for overweight and obesity, which are strongly related to type 2 diabetes and have been positively associated with the risk of liver cancer [41, 42]. Such an association is partially or largely due to hyperinsulinemia and diabetes in overweight subjects, and may therefore represent an overadjustment. Further, history of diabetes and use of antidiabetic drugs was self-reported, and only a few subjects reported information on duration of diabetes. Our findings, however, were in agreement with those reported in other case-control and cohort studies. Moreover, self-reported history of diabetes has been reported to be satisfactorily reliable [43] and the overall prevalence of diabetes among controls is consistent with estimates from national population-based surveys [44]. Finally, concerns have been recently raised about a possible link between long-acting insulin analogue (glargine) and increased risk of selected cancers [39]. Information on the type of insulin was available for 41 out of 51 patients reporting insulin use. Among them, 19 used rapid-acting (e.g., insulin aspart, insulin lispro, and insulin glulisine) or regular/short-acting insulin, 9 used intermediate-acting (e.g., NPH) or long-acting insulin (e.g., insulin detemir, insulin glargine), and the remaining 13 patients used different types of insulin simultaneously. In particular, only 7 patients used insulin glargine, alone or in combination with other types of insulin. Therefore, numbers were too limited to analyze the different insulin types separately.

## 5. Conclusions

Our study confirms the over 2-fold excess risk of HCC in diabetics and suggests that use of metformin may reduce the risk of HCC, while insulin may increase the risk. The diabetes-related excess risk is even greater in diabetic subjects who are also tobacco smokers, the two factors combined yielding an over 6-fold increased risk. The attributable risk in this population is 17% for diabetes alone and 37% for diabetes and tobacco combined. This is an additional reason confirming the importance of stopping smoking in diabetics.

## Figures and Tables

**Table 1:** Distribution of 224 cases of hepatocellular carcinoma (HCC) and 389 controls according to selected factors (Italy, 2005–2012).

	HCC cases		Controls	
	*N*	(%)	*N*	%
Age (years)				
<60	44	(19.6)	154	(39.6)
60–69	81	(36.2)	104	(26.7)
≥70	99	(44.3)	131	(33.7)
Sex				
Male	162	(72.3)	240	(61.7)
Female	62	(27.7)	149	(38.3)
Tobacco smoking^a^				
Never smoker	86	(39.1)	208	(54.2)
Ever smoker	134	(60.9)	176	(45.8)
Hepatitis^a,b^				
No	65	(29.0)	269	(97.1)
Yes	159	(71.0)	11	(2.9)

^a^The sum does not add up to the total because of some missing values.

^b^Hepatitis includes hepatitis B and/or C.

**Table 2:** Distribution^a^ of 224 hepatocellular carcinoma (HCC) cases and 389 controls, and corresponding odds ratios (OR) and 95% confidence intervals (CI), according to history of diabetes (Italy, 2005–2012).

	HCC cases	Controls	OR^b^ (95% CI)
	*N* (%)	*N* (%)	
Diabetes			
No	154 (69.1)	332 (86.5)	1^c^
Yes	69 (30.9)	52 (13.5)	2.25 (1.42–3.56)
Years of diabetes			
<10	9 (5.1)	7 (2.0)	2.96 (0.92–9.45)
≥10	12 (6.9)	3 (0.9)	5.33 (1.34–21.10)
Oral antidiabetic drugs			
Nonusers	46 (20.6)	26 (6.8)	1^c^
Users	23 (10.3)	26 (6.8)	0.51 (0.21–1.28)
Metformin			
Nonusers	57 (25.6)	36 (9.4)	1^c^
Users	12 (5.4)	16 (4.2)	0.44 (0.15–1.27)
Sulfonylureas			
Nonusers	58 (26.0)	43 (11.2)	1^c^
Users	11 (4.9)	9 (2.3)	0.88 (0.24–3.19)
Insulin			
Nonusers	36 (16.1)	34 (8.9)	1^c^
Users	33 (14.8)	18 (4.7)	1.90 (0.74–4.88)

^a^The sum does not add up to the total because of some missing values.

^b^Estimates adjusted for age, sex, tobacco smoking, and alcohol drinking.

^c^Reference category.

**Table 3:** Distribution^a^ of 224 hepatocellular carcinoma (HCC) cases and 389 controls and corresponding odds ratios^b^ (OR) and 95% confidence intervals (CI), according to the combination of smoking and diabetes (Italy, 2005–2012).

Tobacco smoking	History of diabetes	
	Cases : controls, OR (95% CI)	
	No	Yes
Never	62 : 178	24 : 29
	1^c^	1.54 (0.79–2.99)

Ever	90 : 154	44 : 21
	1.82 (1.14–2.90)	6.61 (3.31–13.25)

^a^The sum does not add up to the total because of some missing values.

^b^Estimates adjusted for age, sex, and alcohol drinking.

^c^Reference category.
